# Hepatoprotective activity of *Eucalyptus camaldulensis* extract in murine malaria mediated by suppression of oxidative and inflammatory processes

**DOI:** 10.3389/fcimb.2022.955042

**Published:** 2022-08-12

**Authors:** Hossam M. A. Aljawdah, Rewaida Abdel-Gaber, Esam M. Al-Shaebi, Felwa A. Thagfan, Saleh Al-Quraishy, Mahmood A. A. Qasem, Mutee Murshed, Mohammed M. Mares, Tahani Al-Otaibi, Maysar Abu Hawsah, Mohamed A. Dkhil

**Affiliations:** ^1^ Department of Zoology, College of Science, King Saud University, Riyadh, Saudi Arabia; ^2^ Department of Biology, College of Science, Princess Nourah Bint Abdulrahman University, Riyadh, Saudi Arabia; ^3^ Department of Science and Technology, Al-Nairiyah University College, University of Hafr Al-Batin, Hafr Al-Batin, Saudi Arabia; ^4^ Department of Zoology and Entomology, Faculty of Science, Helwan University, Cairo, Egypt

**Keywords:** *Eucalyptus camaldulensis*, malaria, mice, inflammation, oxidative damage, gene expression

## Abstract

Herbal extracts are promising agents against various parasitic diseases, such as malaria. This study aimed to evaluate the ameliorative action of *Eucalyptus camaldulensis* extract (ECE) against hepatic damage caused by *Plasmodium chabaudi* infection. Mice were allocated into five groups as follows: two groups served as the control non-infected groups that received distilled water and ECE, respectively; subsequent three groups were infected with 10^6^ *P. chabaudi* parasitized erythrocytes; the last two groups were infected with the parasite and then treated with ECE and chloroquine. On day 8 post-infection, the parasite count increased inside erythrocytes (59.4% parasitemia in the infected group). Parasitemia was successfully reduced to 9.4% upon ECE treatment. Phytochemical screening using GC mass spectrometry revealed that ECE contained 23 phytochemical components. Total phenolics and flavonoids in ECE were 104 ± 2 and 7.1± 3 µg/mL, respectively, with 57.2% antioxidant activity. ECE ameliorated changes in liver histopathology and enzymatic activity of alanine aminotransferase, aspartate aminotransferase, and alkaline phosphatase. In addition, ECE prevented oxidative damage induced by the parasite in the liver, as evidenced by the change in the liver concentrations of glutathione, nitric oxide, malondialdehyde, and catalase. Moreover, ECE was able to regulate the expression of liver cytokines, interleukins-1β and 6, as well as IFN-γ mRNA. ECE possesses antiplasmodial, antioxidant, and anti-inflammatory activity against liver injury induced by the parasite *P. chabaudi*.

## Introduction

Malaria is the most common parasitic disease in humans, and is transmitted through the bite of an infected female *Anopheles* mosquito. It remains a major cause of illness and death worldwide where 229 million cases and 409,000 deaths were estimated in 2019 (WHO, 2020).

Malaria pathogenesis research is primarily conducted using rodent malaria parasites such as *Plasmodium chabaudi*, owing to its genome sequence and pathological similarities to the human parasite (Carlton et al., 2002).

Consequently, new, affordable, and effective treatment strategies are needed to reduce the incidence of malaria in endemic countries. Natural products, such as medicinal plants, have long been used as promising and reliable sources for treating parasitic infections, including malaria. The structural diversity of natural products and their ability to interact with therapeutic targets justify their use in the search for new drugs. More than 40% of the authorized drugs on the market are of natural or semi-synthetic origin (Newman and Cragg, 2016). Malaria has been treated with medicinal plants since ancient times. Hence, plant sources could be useful in discovering potential candidates for new antimalarial drugs (Taherkhani et al., 2013).

*Eucalyptus camaldulensis* belongs to the family Myrtaceae and is considered a source of biologically active compounds (Ghalem and Mohamed, 2014). The leaf extract of *E. camaldulensis* (ECE) is known to possess antioxidant (Singab et al., 2011), antimicrobial (Ghalem and Mohamed, 2008), larvicidal (Medhi et al., 2010), pesticidal (Batish et al., 2008), and antiparasitic properties (Hassani et al., 2013). Hence, this study aimed to investigate the antiplasmodial and hepatoprotective properties of ECE against blood-stage malaria caused by *P. chabaudi* in mice by evaluating their oxidative and inflammatory status.

## Materials and methods

### Preparation of *Eucalyptus camaldulensis* extracts

Leaves were collected from plants grown in Qassim, Saudi Arabia (26°04’57.0”N, 43°39’42.2”E). The plant was identified by a specialist at the herbarium of King Saud University. The leaves were air-dried and then ground into a powder. The constituents of the powdered leaves were extracted with 70% methanol (Lubbad et al., 2015). ECE was diluted with distilled water and used for subsequent experiments.

### Determination of total phenolics in ECE

The total phenolic content of ECE was determined using a modified Folin-Ciocalteu method in which 12.5 µL of ECE (1 mg/mL) was mixed with 125 µL of Folin–Ciocalteu reagent (25%) then incubated for 5 min. After that, Na_2_CO_3_ was added and the mixture was kept in dark for 1.5 h. At 760 nm, samples were measured using a microplate reader (Thermo Fisher Scientific, Waltham, MA, USA). A gallic acid standard curve was used to calculate the total phenolic content. (Al-Zharani et al., 2019).

### Determination of total flavonoids in ECE

The total flavonoid content was determined using a colorimetric assay with aluminium chloride. This is by mixing 100 µL of ECE (1 mg/mL) with 100 µL of aluminum chloride (2%). In brief, 100 µL of ECE (1 mg/mL) and 100 µL of aluminum chloride (2%). The absorbance was measured at 368 nm. The flavonoids in the samples were estimated using a calibration curve of quercetin, a standard flavonoid (Ghosh et al., 2013).

### Gas chromatography-mass spectrometry analysis for ECE

The phytochemical analysis of ECE was performed according to the protocol recommended by Kanthal et al. (2014). Gas chromatography-mass spectrometry (GC-MS) was performed using a 7000D GC/MS Triple Quad GC-MS unit (Agilent Technologies, USA).

### DPPH radical scavenging method for antioxidant activity

The free radical scavenging activity of extracts was determined using 2,2-diphenyl-1-picrylhydrazyl (DPPH). In brief, 20 µL of ECE (1 mg/mL) was mixed with 80 µL of a methanolic solution of DPPH (100 mM) and then incubated in the dark for 30 min at 25°C. Absorption was read at 517 nm and radical scavenging activity was calculated (Ghosh et al., 2013).

### Animals and infection

Female C57BL/6 mice (9–11 weeks old) were bred and provided standard diet and water ad libitum. The experiments were approved by the Research Ethics Committee for Laboratory Animal Care at King Saud University (approval no.: KSU-Se-21-77).

Cryopreserved *P. chabaudi* parasites were passaged in donor C57BL/6 mice and intraperitoneally injected into experimental mice as previously described (Wunderlich et al., 1982). A Neubauer chamber was used to calculate the injected dose (10^6^ parasitized erythrocytes). The *P. chabaudi*-parasitized erythrocytes were injected intraperitoneally into mice in 100 µL phosphate buffer (Wunderlich et al., 1982). To determine *P. chabaudi* induced parasitemia, Giemsa-stained smears from tail blood of mice were prepared.

### Experimental design

To determine the suitable dose and peak parasitemia level, the mice were divided into six groups (n = 10). The first group consisted of uninfected control mice that were given only water by gavage. The second to the seventh groups were infected intraperitoneally with 1 × 10^6^ P*. chabaudi*–infected erythrocytes. After 1 h, the third, fourth, and fifth groups of mice were administered ECE at doses of 100, 200, and 400 mg/kg daily for 8 days. The last group was administered 10 mg/kg chloroquine phosphate (CQ) (Sigma-Aldrich, St. Louis, USA) daily for 4 days (Abay et al., 2015). Based on parasitemia results (**Figure S1**), we studied the effect of ECE by repeating the experiment using five groups of mice (n = 5/group): group 1, non-infected control group; group 2, non-infected treated group (treated with 100 mg/kg ECE); group 3, infected group; group 4, infected group + 100 mg/kg ECE; group 5, infected group + 10 mg/kg chloroquine. The dose of ECE was selected based on the study by Anigboro et al. (2020) and results of a preliminary experiment (**Figure S1**). All animals were CO_2_ asphyxiated and dissected on day 8 post infection for sample collection.

### Sample collection

Heparinized tubes were used to collect the blood from the heart. The livers of mice were isolated and cut into small pieces. Parts were preserved in neutral buffered formalin for histopathological examination while others were stored at -80°C to study the antioxidant activity of *E. camaldulensis*, and to extract RNA for the subsequent study of gene expression.

### Biochemical studies

Blood plasma was separated and kept at -20°C until use. Plasma was then analyzed according to the manufacturer’s instructions using commercial kits (Biomerieux, Marcy l’Etoil, France) for alanine aminotransferase (ALT), aspartate aminotransferase (AST), and alkaline phosphatase (ALP).

### Histopathological studies

Freshly prepared liver samples were fixed in 10% neutral buffered formalin and then embedded in paraffin. Hematoxylin and eosin were used for staining after the samples were cut into 5 µm thick sections (Drury and Wallington, 1980). The liver histological scoring was carried out in accordance with the guidelines outlined by Ishak et al. (1995). In summary, this quantification measure for determining the histopathological changes in the liver is based on the examination of liver sections at various magnifications, yielding scores of 1-3, 4-8, 9-12, and 13-18 for minimal, mild, moderate, and severe liver injury, respectively.

### Liver oxidative stress

Malondialdehyde (MDA) was measured in liver homogenates using thiobarbituric acid (Ohkawa et al., 1979). Nitric oxide (NO), glutathione (GSH), and catalase levels were measured as described by Ellman (1959); Green et al. (1982), and Aebi (1984).

### Gene expression

To carry out real-time quantitative polymerase chain reaction (RT-qPCR), liver RNA was isolated using TRIzol (QIAGEN, Hilden, Germany). DNase was used to prepare RNA (Applied Biosystems, Darmstadt, Germany), and a reverse transcription kit was used to convert the samples into cDNA (QIAGEN, Hilden, Germany). The ABI Prism 7500HT sequence detection system (Applied Biosystems, Darmstadt, Germany) was used for PCR along with SYBR green PCR master mix (QIAGEN, Hilden, Germany). Levels of interleukin-1*β* (*IL-1β*), interleukin-6 (*IL-6*), and interferon gamma (*INF-γ*) were investigated using primers from Macrogen Inc. (Seoul, South Korea) (**Table 1**). PCR was performed as described by Dkhil et al. (2019). The fold change in mRNA expression was determined using the 2^−ΔΔCT^ method (Livak and Schmittgen, 2001).

**Table 1:** Primer sequencing used in PCR for cytokines genes.

Gene	Type	Primer sequence (5’ →3’)
IL-1β	Forward Reverse	TGCCACCTTTTGACAGTGATG TTCTTGTGACCCTGAGCGAC
IL-6	Forward Reverse	CTGCAAGAGACTTCCATCCAG AGTGGTATAGACAGGTCTGTTGG
IFN- *γ*	Forward Reverse	CGAAGCAGATGAATCCGCTGA TGCGTGGAAATTGGGTGTCC
GAPDH	Forward Reverse	CCCTTAAGAGGGATGCTGCC ACTGTGCCGTTGAATTTGCC

### Statistical analysis

Significance was evaluated using one-way analysis of variance, and statistical comparisons between the groups were performed using Duncan’s test using a statistical package program (SPSS version 17.0). All values are expressed as the mean and standard error of the mean. All *p*-values were two-tailed, and p ≤ 0.05 was considered significant for all statistical analyses.

## Results

The total phenolic and flavonoid contents in ECE reached 104 ± 2 and 7.1± 3 µg/mL, respectively while the antioxidant activity of ECE reached 57.2% (**Table 2**).

**Table 2:** Total phenolic, flavonoid contents and radical scavenging activity of *E. camaldulensis* extract.

Total phenolics (µg/g)	Total flavonoid (µg/g)	Antioxidant (%)
104 ± 2	7.1 ± 0.7	57.2

**Figure 1:**
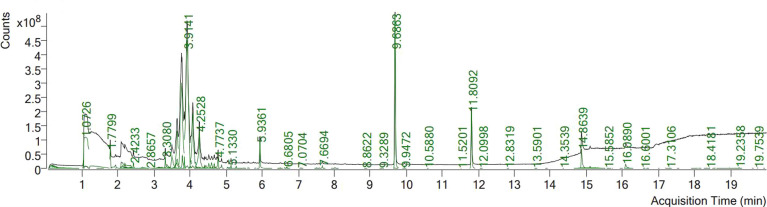
GC-MS chromatogram of *Eucalyptus camaldulensis* leaf extracts.

Phytochemical screening using GC mass spectrometry revealed that ECE contained 23 phytochemical components (**Table 3**, **Figure 1**). The compounds benzene, 1,2,4,5-tetramethyl-, naphthalene, 2,4-Di-tert-butylphenol, hexadecanoic acid, methyl ester (palmitic acid), methyl stearate, hexadecanoic acid, and 2-hydroxy-1- (hydroxymethyl)ethyl ester appeared in **Figure 1** with elevated peaks.

**Table 3:** Identification of phytochemical compounds by GC-Mass in *Eucalyptus camaldulensis* leaf extracts.

Component RT	Compound Name	Molecular Wight	[M-H]- (m/z)Molecular Wight -1	Formula	area	Peak %
3.9141	Benzene, 1,2,4,5-tetramethyl-	134.2182	133.2182	C_10_H_14_	2366033968	53.48
4.2528	Naphthalene	128.1705	127.1705	C_10_H_8_	417661948	9.44
4.7737	Naphthalene, 2-methyl-	142.1971	141.1971	C_11_H_10_	65276121	1.47
5.1330	Cyclooctane, 1,4-dimethyl-, trans-	140.2658	139.2658	C_10_H_20_	14053670	0.32
5.9361	2,4-Di-tert-butylphenol	206.3239	205.3239	C_14_H_22_O	113319951	2.56
6.6805	Epizonarene	204.3511	203.3511	C_15_H_24_	7489114	0.17
7.0704	1-Naphthalenol, 5,6,7,8-tetrahydro-2,5- dimethyl-8-(1-methylethyl)-	218.3346	217.3346	C_15_H_22_O	1707837	0.037
7.6694	Tridecanoic acid, 12-methyl-, methyl ester	242.3975	241.3975	C_15_H_30_O_2_	10127962	0.23
8.8622	Benzenamine, N,N,3-trimethyl-	135.2062	134.2062	C_9_H_13_N	365918	0.007
9.3289	Methyl p-(2-phenyl-1-benzimidazolyl)benzoate	328.4	327.4	C_21_H_16_N_2_O_2_	1867052	0.011
9.6863	Hexadecanoic acid, methyl ester (Palmitic acid)	270.4507	269.4507	C_17_H_34_O_2_	889708992	20.12
9.9472	Benzenepropanoic acid, 3,5-bis(1,1- dimethylethyl)-4-hydroxy-, methyl ester	292.4131	291.4131	C_18_H_28_O_3_	6432461	0.15
10.5880	Glycine, 2-cyclohexyl-N-(but-2-yn-1- yl)oxycarbonyl-, but-2-yn-1-yl ester	305.4	304.4	C_17_H_23_NO_4_	160897	0.003
11.5201	2-Furoic acid, 4-chlorophenyl ester	222.62	221.62	C_11_H_7_ClO_3_	103043	0.03
11.8092	Methyl stearate	298.5038	297.5038	C_19_H_38_O_2_	339746120	7.67
12.0998	Thiophene-2-carboxamide, N-methyl-N-(hept2-yl)-			C_13_H_21_NOS	548300	0.012
12.8319	4-Benzoyl-N-(4-methoxy-phenyl)-benzamide	331.4	330.4	C_21_H_17_NO3	559538	0.013
13.5901	4-Amino-2-(4’-cyanobutyl)-5,6-trimethylenepyrimidine	216.28	215.28	C_12_H_16_N_4_	277367	0.007
14.3539	2H-1-Benzopyran-3(4H)-one, 8-methoxy-2- phenyl-, oxime	269.29	268.29	C_16_H_15_NO_3_	416461	0.009
14.8639	Hexadecanoic acid, 2-hydroxy-1- (hydroxymethyl)ethyl ester	330.5026	329.5026	C_19_H_38_O_4_	149010470	3.368
16.0890	Octadecanoic acid, 2,3-dihydroxypropyl ester	358.5558	357.5558	C_21_H_42_O_4_	37076654	0.84
16.6001	3-Methyl-pyrrolo(2,3-b)pyrazine	133.15	132.15	C_7_H_7_N_3_	654333	0.015
17.3106	Propanedinitrile, 2-(5-phenylthio-2- thienylmethylene)-	268.4	267.4	C_14_H_8_N_2_S_2_	1607058	0.038

**Figure 2:**
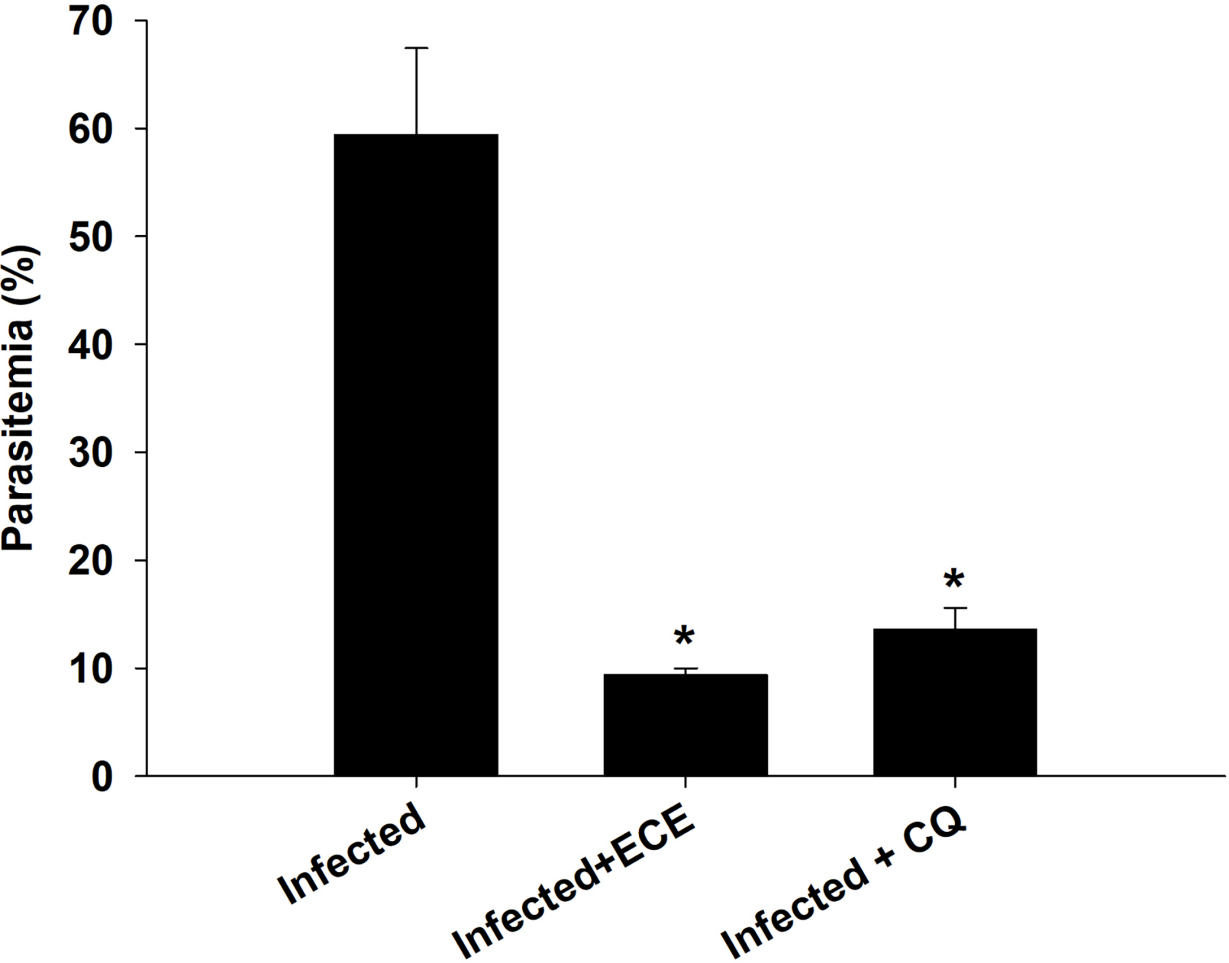
Percentage of parasitemia in mice infected with *P. chabaudi* and treated with *Eucalyptus camaldulensis* extract (ECE) and Chloroquine (CQ). Data are presented as mean ± SD at p ≤ 0.001. *, Significance against infected group.

**Figure 2** shows the parasitemia in the infected (59.4 ± 8.5%) and ECE-treated mice (9.4 ± 0.6%) compared to mice administered the reference drug chloroquine (13.6 ± 2%).

Infection induced a significant increase in the liver enzymes ALT, AST, and ALT (**Table 4**). ECE ameliorated the changes in liver enzymes, similar to that observed after treatment with the reference drug.

**Table 4:** Effect of ECE on ALT, AST and ALP of mice infected with *P. chabaudi*.

Group	ALT (U/L)	AST (U/L)	ALP (U/L)
Control	20 ± 2.4	116 ± 11	93 ± 6
ECE	23.2 ± 1.2	123 ± 11	78 ± 10
Infected	31 ± 4^*^	205 ± 7^*^	16 ± 6^*^
Infected + ECE	22 ± 1^#^	148 ± 6^*#^	24 ± 9*
CQ	16.8 ± 1.5^*#^	112 ± 6^#^	41 ± 4^*#^

Values are mean ± SEM. Significance at p ≤ 0.05 against control (*) and Infected (#) animals.

**Figure 3:**
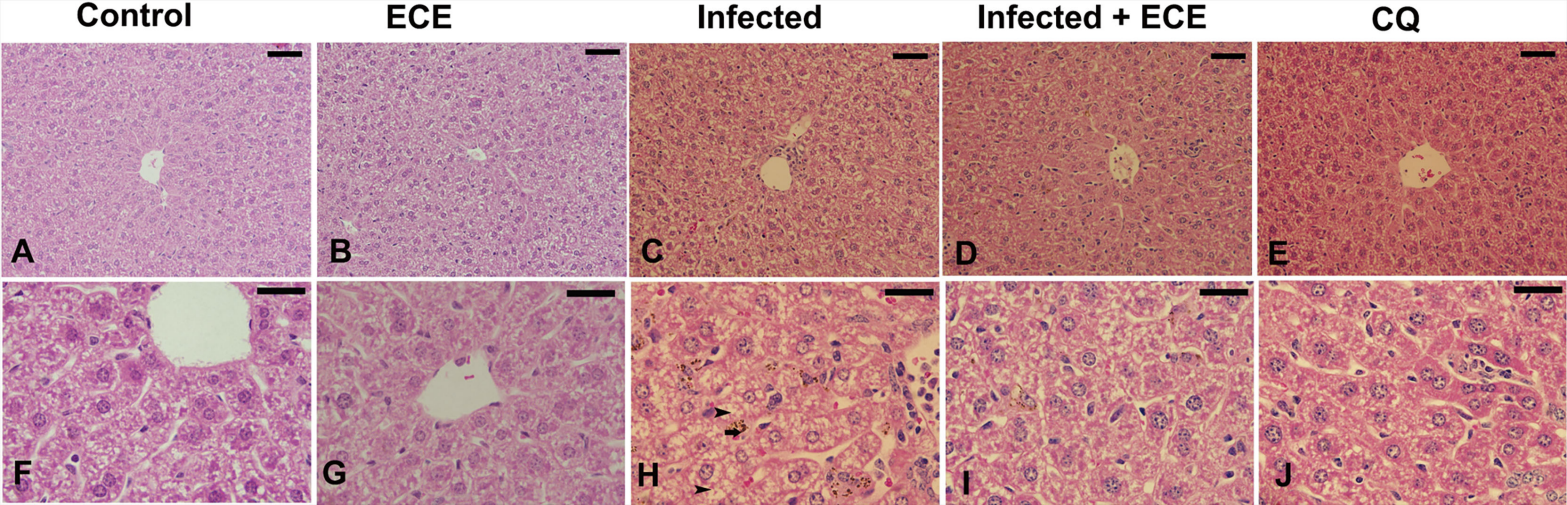
Effect of *Eucalyptus camaldulensis* extract (ECE) on the liver histology of mice infected with *P. chabaudi*. **(A, F)** non-infected group. **(B, G)** ECE treated group. **(C, H)** Infected group with inflammation, malaria pigment (arrow) and hepatocytic vacuolation (arrow head). **(D, I)** Infected-ECE treated group with improved structure. **(E, J)** infected-chloroquine treated group with improved structure. Scale bar = 25 µm.

Mice infected with *P. chabaudi* demonstrated extensive liver damage with inflammatory cells, whereas healthy controls and ECE-administered groups had normal liver architecture. Hepatic lesions were relieved, and the liver score was lowered in ECE-treated mice, which was linked to a reduction in inflammatory cell infiltration (**Figure 3**). Ishak’s score indicated that the infected group’s liver activity index ranged from 13 to 15. When mice were given ECE, the liver index dropped to 5-8 and when treated with chloroquine, the score reached 6-8 (**Table 5**).

**Table 5:** Histology score demonstrating the protective effect of *Eucalyptus camaldulensis* extract (ECE) against *P. chabaudi*-parasitized erythrocytes in the liver of mice compared to chloroquine (CQ).

Group	Modified histological Score of Ishak	Microscopic observation					
		Apoptosis or Necrosis	Hemorrhage	Inflammation	Hyperplasia of Kupffer cells	Cell swelling
**Control**	2	+	0	0	0	0
**ECE**	2	+	0	0	0	0
**Infected**	13-15	+++	+++	+++	+++	++
**Infected + ECE**	5-8	+	+	++	+	+
**CQ**	6-8	+	+	++	+	0

0, absent; +, mild; ++, moderate; and +++, severe.

**Figure 4:**
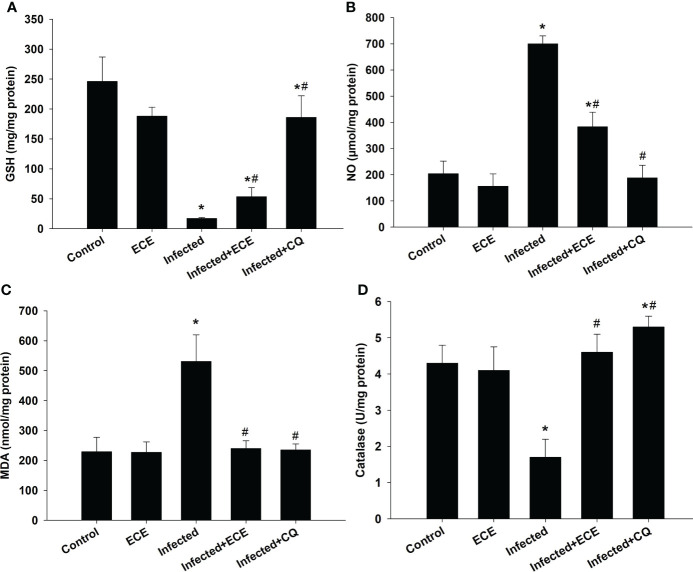
Effect of *Eucalyptus camaldulensis* extract (ECE) on the level of **(A)** glutathione (GSH), **(B)** nitric oxide (NO), **(C)** malondialdehyde (MDA), and **(D)** catalase in liver of mice infected with *P. chabaudi*. Data are presented as mean ± SD at p ≤ 0.001. * and #: Significance against the control and infected groups, respectively.

Compared to those in the control group, mice in the infected group exhibited significantly higher liver MDA and NO levels, lower GSH levels, and a significant decrease in antioxidant catalase enzyme activity (**Figure 4**). Notably, ECE as CQ supplementation restored the balance between oxidants and antioxidants in the hepatic tissue following infection by decreasing the production of MDA and NO and enhancing the examined antioxidant proteins (**Figure 3**).

**Figure 5:**
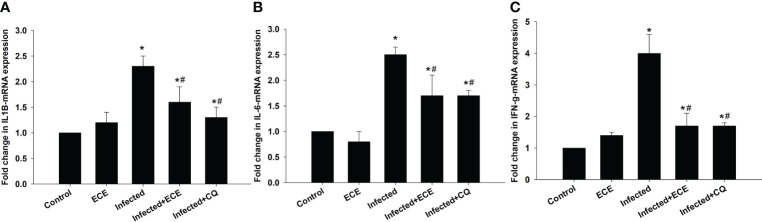
Effect of *Eucalyptus camaldulensis* extract (ECE) on **(A)** IL1β, **(B)** IL-6, and **(C)** IFN-γ-mRNA expression in liver of mice infected with *P. chabaudi*. Data are presented as mean ± SD at p ≤ 0.001. * and #: Significance against the control and infected groups, respectively.

*P. chabaudi* infection induced hepatic tissue inflammation, as reflected in a significant elevation (p < 0.05) in the pro-inflammatory cytokine levels of IL-1β, IL-6, and IFN-*γ* (**Figure 5**). However, this inflammatory response was significantly suppressed following ECE or CQ administration, reflecting the protective impact of ECE as CQ against inflammatory events triggered during the development of malaria.

## Discussion

Natural products play a crucial role in the exploration of new leads for drug therapy against human diseases. Most medicinal agents used in the treatment of malaria are extracted from plants or natural products (Moyo et al., 2020). Moreover, there is always an urgent and continuing call to look for new antimalarial agents a most antimalarial drugs in current use have been rendered inefficient due to drug resistance.

*Eucalyptus camaldulensis* is considered a source of biologically active compounds rich in phenolics and flavonoids. These compounds could be the contributing factors towards the antimalarial effect and reduced parasitemia following the treatment of infected animals (**Table 2**). Recently, Anigboro et al. (2020) reported that *E. camaldulensis* aqueous leaf extracts could protect against *P. berghei* by significantly reducing malarial-induced physiological imbalances in liver and renal biomarkers, as well as serum electrolytes and this is owing to the presence of phytochemically active compounds in the extract by inducing malaria and dysfunction in the liver and kidney.

The GC mass analysis showed that ECE possesses several active compounds, including Naphthalene, an intestinal antiseptic and vermicide (IARC Working Group on the Evaluation of Carcinogenic Risks to Humans, 2002) as well as hexadecanoic acid and methyl ester (palmitic acid) are used in herbal medicine as antioxidant, anti-inflammatory, and anti-apoptotic agents (Hamed et al., 2020). In addition, methyl stearate is used to treat neural and cardiac diseases (Chen et al., 2020).

In our study, ECE treatment improved the health of host mice by reducing both weight loss and anemia relative to non-infected controls due to decreased parasitemia, which causes loss of appetite and decrease in erythrocyte count and hemoglobin content during infection. Similar results were obtained in our previous studies using different medicinal plants (see Dkhil et al., 2021).

*Eucalyptus camaldulensis* can generally reduce the activity of liver function enzymes (Anigboro et al., 2020). In this study, treatment of *P. chabaudi*-infected animals with ECE decreased blood plasma ALT, AST, and ALP levels. This demonstrates that plant extracts can improve the effects of malaria-induced liver damage.

The observed potential therapeutic effect of the extracts on liver enzyme activity could be attributed to parasite elimination and/or antioxidant effects of the plant extracts, which could be derived from its bioactive constituents (Gomes et al., 2011; Nutham et al., 2015).

The decrease in erythrocyte count and hemoglobin concentrations in *P. chabaudi*-infected mice indicated that the mice were anemic (Dkhil et al., 2019). Anemia causes changes in tissue physiology and histology, oxidative damage, and formation of free radicals. According to Omodeo-Salè et al. (2003), oxidative damage promotes the development of anemia during *P. falciparum* infection. This is due to an imbalance between oxidants and antioxidants, resulting in the formation of free radicals that cause oxidative injury to the liver (Guha et al., 2006). In addition, anemia is due to parasite usage of hemoglobin as a source of nutrition, thereby liberating heme, which promotes oxidative damage and produces histopathological changes in the liver (Kumar and Bandyopadhyay, 2005). These biochemical and histological findings are consistent with those of previous studies (Lubbad et al., 2015; Dkhil et al., 2019) that investigated the hepatoprotective effects of herbal extracts against *P. chabaudi* infection.

Our findings indicated that *P. chabaudi*-induced hepatic inflammation is accompanied by an inflammatory reaction in the liver. Furthermore, our results indicate that ECE not only kills *Plasmodium* parasites in animals but also has anti-inflammatory properties that protect the liver.

Infection with *P. chabaudi* could produce an inflammatory reaction in the mouse liver. This response is characterized by a change in liver structure as well as significant changes in oxidative stress biomarkers (GSH, NO, MDA, and catalase) and the inflammatory cytokines IFN-, IL-1β, and IL-6. *Plasmodium* infection is characterized by early and intensive cytokine-mediated effector mechanisms that kill or eliminate parasite-infected cells, and are linked to both acquired and innate immune responses (Gowda and Wu, 2018). In addition, an increase in IL-6 and IFN-γ levels is associated with hyperparasitemia (Angulo and Fresno, 2002). Moreover, inflamed liver cells, activated leukocytes, and macrophages generate excessive levels of pro-inflammatory cytokines and enzymes under inflammatory conditions, assisting in the initiation of the innate immune response (Wunderlich et al., 2014). IL-1 and IL-6 are proinflammatory cytokines that play a big role in inflammation and immunity, and they have been discovered to be upregulated following *Plasmodium* infection (Dkhil et al., 2021). Interestingly, ECE administration inhibited the development of inflammatory reactions following *P. chabaudi* infection by downregulating cytokines and controlling oxidative changes induced in the liver. All of these reduced inflammatory responses of ECE was originally due to the reduced number of parasitized erythrocytes as seen in **Figure 1**. Several reports support our findings that medicinal plants are able to reduce the parasitemia and protect the liver from inflammation (Wunderlich et al., 2014; Dkhil et al., 2019; Dkhil et al., 2021).

Here, we postulate that *E. camaldulensis* is capable of protecting the hepatic tissue following malaria-induced infection, as evidenced by the improved percentage of parasitemia and histological structure. In addition, *E. camaldulensis* restored the balance between oxidants and antioxidants, while suppressing inflammation in the liver. These findings indicate that *E. camaldulensis* is a promising hepatoprotective agent against malaria. Furthermore, additional studies are necessary to clarify the mechanisms underlying the liver’s capacity to respond to both *P. chabaudi* and *E. camaldulensis*.

## Data availability statement

The original contributions presented in the study are included in the article/**Supplementary Material**, further inquiries can be directed to the corresponding author.

## Ethics statement

The experiments were approved by the Research Ethics Committee for Laboratory Animal Care at King Saud University (approval no.: KSU-Se-21-77).

## Author contributions

HA, SA-Q, and MD designed the study; HA, RA-G, EA-S, FT, MQ, MM, MMM, TA-O, MA, and MD carried out the experiments and analyzed the data. All authors wrote and revised the manuscript. All authors read and approved the final manuscript.

## Funding

This study was supported by Researchers Supporting Project (RSP2021/03), King Saud University, Riyadh, Saudi Arabia.

## Conflict of interest

The authors declare that the research was conducted in the absence of any commercial or financial relationships that could be construed as a potential conflict of interest.

## Publisher’s note

All claims expressed in this article are solely those of the authors and do not necessarily represent those of their affiliated organizations, or those of the publisher, the editors and the reviewers. Any product that may be evaluated in this article, or claim that may be made by its manufacturer, is not guaranteed or endorsed by the publisher.
