# Supplementary figures and images for: Hepatoprotective activity of Eucalyptus camaldulensis extract in murine malaria mediated by suppression of oxidative and inflammatory processes
Source: Front Cell Infect Microbiol. 2022 Aug 12;12:955042. doi: 10.3389/fcimb.2022.955042 (PMC9412018; doi:10.3389/fcimb.2022.955042)

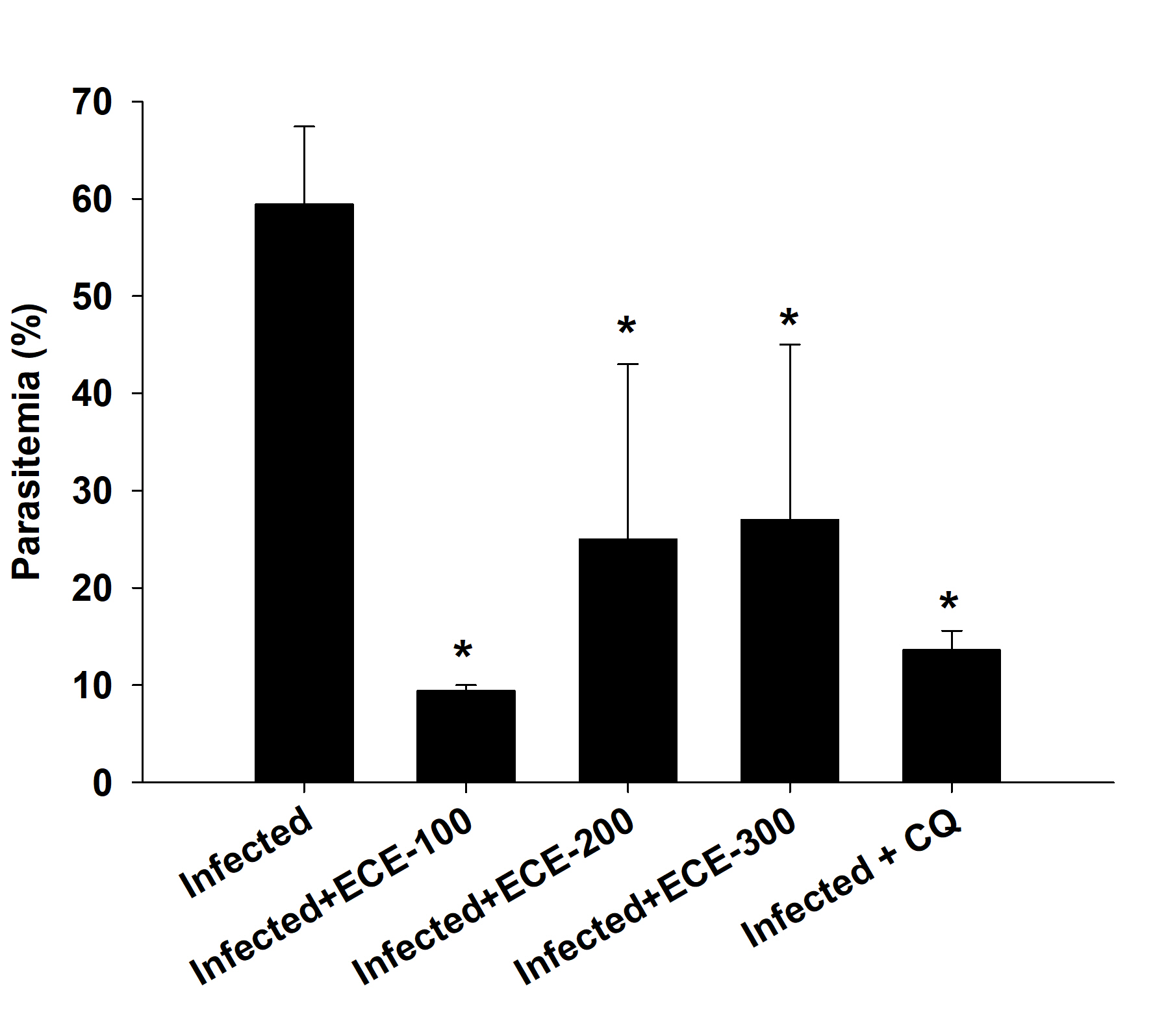

Supplement: Supplementary file 1 [file Image_1.jpeg]
